# Influence of physical exercise on activities of daily living in older adults: an empirical analysis based on propensity score matching and difference-in-differences

**DOI:** 10.3389/fpubh.2025.1463348

**Published:** 2025-04-17

**Authors:** Xiaoni Ma, Xiaotian Li, Li Che, Jie Dong

**Affiliations:** ^1^Graduate School, Capital University of Physical Education and Sports, Beijing, China; ^2^School of Recreation and Community Sport, Capital University of Physical Education and Sports, Beijing, China

**Keywords:** activities of daily living, exercise, COVID-19, difference-in-differences model, propensity score matching, older adults

## Abstract

**Background:**

Activities of Daily Living (ADL) are crucial for assessing older adult’s ability to live independently. Physical exercise has a positive impact on ADL. During the COVID-19 pandemic, the reduction of social interaction and the limited use of exercise facilities led some older adults to reduce regular exercise and show more sedentary behavior. This study aimed to examine the influence of physical exercise on the Activities of Daily Living (ADL) among older adults during the COVID-19 pandemic.

**Methods:**

Using the China Longitudinal Aging Social Survey (CLASS) data, propensity score matching (PSM) was performed on the sample, with the interaction between participation in physical exercise and observation year as the core independent variables, and instrumental activities of daily life (IADL) and basic activities of daily life (BADL) as the dependent variables for difference-in-differences (DID) regression analysis. Verify age heterogeneity through grouped regression, and use mediation effect analysis to examine the role of retirement.

**Results:**

In the context of COVID-19, participation in physical exercise had a positive impact on the IADL and BADL of older adults. The IADL and BADL of older adults who participated in physical exercise were 0.189 and 0.119 units higher than those who did not participate in physical exercise. This positive impact also varied by age, for older adults aged 75 years and above, participation in physical exercise exerted a significant positive impact on both IADL and BADL. In contrast, among those under 75 years of age, no significant effects of physical exercise on IADL or BADL were detected. The analysis further revealed retirement status emerged as a significant masking variable that amplifies observed differences in ADL outcomes when controlled analytically.

**Conclusion:**

During the pandemic, physical exercise still has a positive impact on the IADL and BADL of the older adults. The older adults aged 75 and above are more reliant on physical exercise to improve their IADL and BADL. Due to the survivor effect, the relative negative effect of retirement on the IADL and BADL of the older adults who engage in physical exercise is more pronounced.

## 1 Introduction

With the ongoing demographic shifts in China, the aging population is increasing. By 2022, individuals aged 60 and above constituted 19.8% of the total population (1). With advancing age, there is a notable decline in physical abilities, especially immune function, among older adults (2). This deterioration in immune function is linked to various health issues, severely threatening their overall well-being. Accordingly, addressing healthy aging is an urgent issue. Activities of daily living (ADL) is a critical indicator for assessing the independent living capability of older adults (3). ADLs are categorized into two types: Instrumental Activities of Daily Living (IADL) and Basic Activities of Daily Living (BADL). IADL involves more complex tasks related to managing one’s environment, such as transportation, shopping, financial management, cooking, and housekeeping (4). On the other hand, BADL consists of basic physical tasks necessary for preserving independence, such as eating, dressing, transferring in and out of bed, toileting, bathing, and other fundamental self-care activities (4).

Declines in IADL and BADL can reduce the scope of activities for the older adults, decrease their contact with the outside world, and adversely affect their physical and mental health (5). An observational study conducted in France over 5 years revealed that most older adults first experience a decline in physical activity capacity as a reduction in IADL, followed by challenges in BADL (6). The emergence of the Coronavirus disease 2019 (COVID-19) pandemic in late 2019 has led serious global health crisis, impacting socio-economic development and public safety due to its high transmission rate and widespread nature. Older adults are particularly at risk of severe illness and mortality from the virus when compared to younger populations (7).

Existing research has shown that physical exercise plays a crucial role in promoting physical health, reducing the negative effects of certain diseases, and supporting positive aging by positively influencing IADL and BADL (8–12). However, most studies focus on standard conditions, rather than during pandemics. During the COVID-19 pandemic, older adults, as a vulnerable population, should pay more attention to maintaining and improving their health.

In addition, during this period, the reduction of social interaction and the limited use of exercise facilities led some older adults to reduce regular exercise (13) and show more sedentary behavior (14). A meta-analysis has shown that during the COVID-19 pandemic, the daily sedentary time for the older adults increased by 46.9 ± 22.0 min (15). Studies also indicate that the longer older adults remain sedentary, the more likely they are to experience insufficient ADL, regardless of the time they spend engaging in moderate to vigorous activities (16). Thus, even substantial daily doses of moderate to vigorous physical activity may not offset the adverse effects of a sedentary lifestyle. This study aims to investigate whether physical exercise during the pandemic still meets the needs of older adults to improve their IADL and BADL. To make up for the shortcomings of existing research. By utilizing the “2020 COVID-19 pandemic” as an exogenous shock, this paper applies propensity score matching (PSM) and difference-in-differences (DID) methodologies to analyze the impact of physical exercise on IADL and BADL under pandemic conditions and examines the heterogeneous effects of physical exercise across different age groups.

## 2 Materials and methods

### 2.1 Study population

Data for this study were collected from the national baseline surveys of the China Longitudinal Aging Social Survey (CLASS) carried out in 2018 and 2022 (17). CLASS is a national, ongoing, large-scale social survey that collects social, economic, family, and health information data of Chinese older adults people aged 60 and older. Its objective is to investigate the challenges faced by the aging population in China and to provide a robust theoretical and empirical foundation for addressing aging-related issues in the country. This survey adopts a stratified multi-stage probability sampling method to select county-level areas (including counties, county-level cities, and districts) as the primary sampling unit, and villages/residents’ committees as the secondary sampling unit. The recruited participants are Chinese citizens aged 60 and above (with no age limit) residing at the current address. The interviewer reads the questions and answers one by one according to the questionnaire, and the interviewee selects the corresponding answer items. Then the interviewer records them on the questionnaire. To date, CLASS has conducted four national follow-up surveys in 2014, 2016, 2018, and 2022. For this study, the data from 2018 and 2022 were selected as they are the periods closest to the emergence of COVID-19.

The initial sample size in 2018 was 11.416, and in 2022 it was 11.398. When constructing a DID model, an experimental group and a control group are required. Therefore, this study removed samples that participated in physical exercise in 2018, resulting in a total sample size of 14,589.

### 2.2 Variables

The variables covered in this study are summarized in Table 1.

**Table 1:** Definition of variables.

Variables	Data information
Outcome variable (activities of daily living)	
Instrumental activities of daily living	Combine the following variables into one variable (1 = can; 2 = cannot): Can you make phone calls by yourself? Can you use public transportation (such as busses and subways) by yourself? Can you go shopping by yourself? Can you manage your finances? Can you take medication by yourself? Can you cook by yourself? Can you do household chores (such as cleaning, doing laundry, washing dishes) by yourself?
Basic activities of daily living	Combine the following variables into one variable (1 = does not need help; 2 = needs some help; 3 = unable to do): Can you dress yourself? Can you bathe yourself (shower or bath)? Can you eat by yourself? Do you experience urinary incontinence? Do you experience fecal incontinence? Can you use the toilet by yourself? Can you go up and down stairs? Can you move around indoors?
Treatment variable	
Participation in physical exercise	What is the frequency of your participation in physical exercise? (Define less than once a month as not participating in physical exercise)
Observation year	2018, 2022
Control variables	
Gender	Select ‘Gender’: 0 = Male; 1 = Female
Age	Survey Year - Birth Year+1
Education level	Select: “What is your cultural level?” 1 = illiterate; 2 = private school/literacy class; 3 = primary school; 4 = high school/vocational school; 5 = associate degree; 6 = Bachelor’s degree or above
Marital status	Select: “What is your current marital status?” 1 = Married with spouse; 2 = Widow; 3 = divorce; 4 = unmarried
Spouse education level	Select: “What is your spouse’s educational level?” 1 = illiterate; 2 = private school/literacy class; 3 = primary school; 4 = high school/vocational school; 5 = associate degree; 6 = Bachelor’s degree or above
Hospitalization frequency	Select: “How many times have you been hospitalized in the past two years?”
Chronic disease comorbidity status	Answer: “How many chronic diseases do you think you have?” (hypertension, heart disease, diabetes or high blood sugar, cerebrovascular disease, kidney disease, liver disease, tuberculosis, cervical/lumbar disease, arthritis or rheumatism, breast disease, reproductive system disease, prostate disease, urinary system disease, glaucoma/cataract, cancer/malignant tumor, osteoporosis, chronic lung disease, nervous system disease, gastroenteritis or digestive system disease, Parkinson’s disease, deafness and other chronic diseases)
Mediating variable	
Retirement	Choice: “Have you applied for retirement?” 1 = Retired; 2 = Employment

### 2.3 Statistical analysis

#### 2.3.1 Identification strategy

The Difference in Differences (DID) method relies on the assumption that treatment and control groups are assigned randomly. However, in natural experiments, observational data is not randomly distributed, leading to a non-random selection process influenced by factors like gender, age, and education level. The Propensity Score Matching-Difference in Differences (PSM-DID) method effectively mitigates this issue, as it satisfies the common trends assumption (18). PSM resolves the self-selection problem of samples by integrating many observable confounding variables into a single variable: the propensity score. Since individuals with similar propensity scores share similar characteristics in other variables, matching the treatment and control groups by propensity scores balance the baseline levels of the two groups, achieving an effect akin to random grouping (19).

In this study, PSM is used to address the sample’s self-selection issue by matching individuals from the control group who are similar to those in the treatment group using PSM (20). Subsequently, the net effects of the treatment are compared through Difference-in-Differences (DID) (21). The fundamental idea of DID is to construct a double difference statistic reflecting the effects of physical exercise by comparing the differences between the control and treatment groups in 2018 and 2022. In this research, DID initially assesses within-group differences to eliminate estimation biases caused by certain unobservable individual factors that remain constant over time; it then evaluates between-group differences to remove the impact of temporal trends. Through these two differences analyses, the effect of the treatment, namely the impact of changes in physical exercise participation status on individual IADL and BADL, is determined.

Using ordinary regression to estimate causal relationships between variables may cause problems. Firstly, the issue of self-selection arises because the decision to engage in physical exercise is a personal choice influenced by individual characteristics (such as age, gender, education level, and the presence of chronic diseases). This can result in “self-selection bias” when comparing participants of physical exercise to non-participants. Secondly, the problem of endogeneity in variables is addressed by DID, where the COVID-19 pandemic is exogenous to micro-individuals, thus avoiding reverse causality issues. Moreover, DID utilizes a fixed effects model, partially addressing the omitted variable bias problem. Considering these factors, this paper opts for the PSM-DID estimate to evaluate the effect of physical exercise on BADL and IADL among the older adults.

Since the onset of the COVID-19 pandemic in December 2019, it has influenced the physical activity behaviors of the older adults. This study designates individuals who did not participate in physical exercise in 2018 but did in 2022 as the experimental group, and those who did not participate in physical exercise in both 2018 and 2022 as the control group. By using the PSM-DID method, this study seeks to assess the influence of physical exercise on the IADL and BADL of the older adults throughout the COVID-19 pandemic.

#### 2.3.2 Empirical model

##### 2.3.2.1 Baseline model

This study aims to explore the effects of participation in physical exercise on individual IADL and BADL against the backdrop of the pandemic, utilizing the classical DID approach to ascertain the net effects of experimental treatments. The DID estimation model employed in this paper is structured as follows:


(1) $$\begin{array}{l} Y_{i,t}=\alpha_{0}+\beta_{1}post_{t}*\mathrm{trea}t_{i}+\beta_{2}X_{i,t}\\ +\mu_{i}+\gamma_{t}+\varepsilon_{i,t}\;\left(i=1,\ldots ,n;\;t=1,2\right) \end{array}$$

In Equation 1, the dependent variable $Y_{i,t}$ represents the older adults’ IADL or BADL. The core explanatory variable of the model is $post_{t}*\mathrm{trea}t_{i}$ , where $post_{t}$ is set to 0 for observations in the year 2018 and to 1 for the year 2022. $\mathrm{trea}t_{i}$ takes a value of 0 when the individual does not participate in physical exercise and a value of 1 when the individual does participate. The model includes control variables $X_{i,t}$ . and incorporates two-way fixed effects: $\mu_{i}$ represents individual fixed effects, $\gamma_{t}$ represents time fixed effects, and $\varepsilon_{i,t}$ is the error term of the model.

The baseline model in this paper primarily includes control variables such as gender, age, education level, marital status, spouse’s education level, retirement status, number of hospital stays, and comorbid chronic conditions (Table 1).

##### 2.3.2.2 Heterogeneity analysis model

The impact of physical exercise may vary across different age groups. To further explore the heterogeneous effects of physical exercise, this paper establishes Equations 2, 3, using the age of 75 as a dividing line. If there is a difference between the coefficients of Equations 2, 3, it indicates that the effects of physical exercise vary between age groups above and below 75. If there are differences between the coefficients of Equations 2, 3, it suggests that the effects of physical exercise differ between the age groups of “under 75 years old” and “75 years old and above.” To verify these differences, the Fisher combined test and “1,000 bootstrap samples” are employed to evaluate the inter-group coefficient differences between the regression results of the “under 75 years old” and “75 years old and above” age groups.


(2) $$\begin{array}{l} Y_{i,t}=\alpha_{0}+\beta_{1}post_{t}*\mathrm{trea}t_{i}+\beta_{2}X_{i,t}+\mu_{i}+\gamma_{t}+\varepsilon_{i,t}\;\\ \mathrm{if}\;\mathrm{age}<75\;\left(i=1,\ldots ,n;\;t=1,2\right) \end{array}$$

(3) $$\begin{array}{l} Y_{i,t}=\alpha_{0}+\beta_{1}post_{t}*\mathrm{trea}t_{i}+\beta_{2}X_{i,t}+\mu_{i}+\gamma_{t}+\varepsilon_{i,t}\;\\ \mathrm{if}\;\mathrm{age}\geq 75\;\left(i=1,\ldots ,n;\;t=1,2\right) \end{array}$$

##### 2.3.2.3 Mediating analysis model

Mediation effects refer to the phenomenon where an independent variable not only affects the dependent variable directly but also has an indirect effect through a mediator variable. To further investigate whether there are mediation effects in the pathways through which physical exercise impacts the IADL and BADL of the older adults, this study, drawing on the methodological summaries of mediation analysis by Wen Zhonglin and others (22), establishes Equations 4, 5.


(4) $$\begin{array}{l} \mathrm{Retiremen}t_{i,t}=\alpha_{2}+\beta_{3}post_{t}*\mathrm{trea}t_{i}+\beta_{4}X_{i,t}\\ +\mu_{i}+\gamma_{t}+\varepsilon_{i,t}\;\left(i=1,\ldots ,n;\;t=1,2\right) \end{array}$$

(5) $$\begin{array}{l} Y_{i,t}=\alpha_{3}+\beta_{5}\mathrm{Retiremen}t_{i,t}+\beta_{6}X_{i,t}\\ +\mu_{i}+\gamma_{t}+\varepsilon_{i,t}\;\left(i=1,\ldots ,n;\;t=1,2\right) \end{array}$$

In Equation 4, the dependent variable is Retirement, and the explanatory variable is $post_{t}*\mathrm{trea}t_{i}$ . In Equation 5, the dependent variable is either IADL or BADL, and the explanatory variable is $\mathrm{Retiremen}t_{i,t}$ . By considering Equations 1, 4, 5 comprehensively, we can test the role of retirement in the impact of physical exercise on the older adults.

## 3 Results

Table 2 shows the situation of IADL and BADL among older adults who did not participate in physical exercise in 2018, and the difference in IADL and BADL between older adults who participated and did not participate in physical exercise in 2022. The results showed that the IADL and BADL of older adults who participated in physical exercise in 2022 were significantly higher than those who did not participate in physical exercise. The IADL and BADL of older adults who did not participate in physical exercise in 2022 were significantly lower than those who did not participate in physical exercise in 2018.

**Table 2:** Comparison of ADL and differences between individuals who participate in physical activity and those who do not participate in physical activity in 2018 and 2022.

	Mean		Differences in exercise in non-exercise (*P*)
	Participation in physical exercise	Non-participation in physical activity	
IADL			
2018	—	20.266	—
2022	20.244	19.954	<0.001
Differences in 2018 and 2022 (*P*)	—	<0.001	—
BADL			
2018	—	26.580	—
2022	26.498	26.376	<0.05
Differences in 2018 and 2022 (*P*)	—	<0.001	—

The sample sizes for participants engaged in physical exercise were 2.203 in 2018 and 4.549 in 2022, while the sample sizes for non-participants in physical exercise were 9.093 in 2018 and 6.849 in 2022.

### 3.1 Propensity score matching

In matching the samples, the status of individual participation in physical exercise in 2018 and 2022 was chosen as the subject of analysis. Variables selected for matching included gender, age, educational level, marital status, spouse’s educational level, retirement status, number of hospital stays, and comorbid chronic conditions.

**Figure 1:**
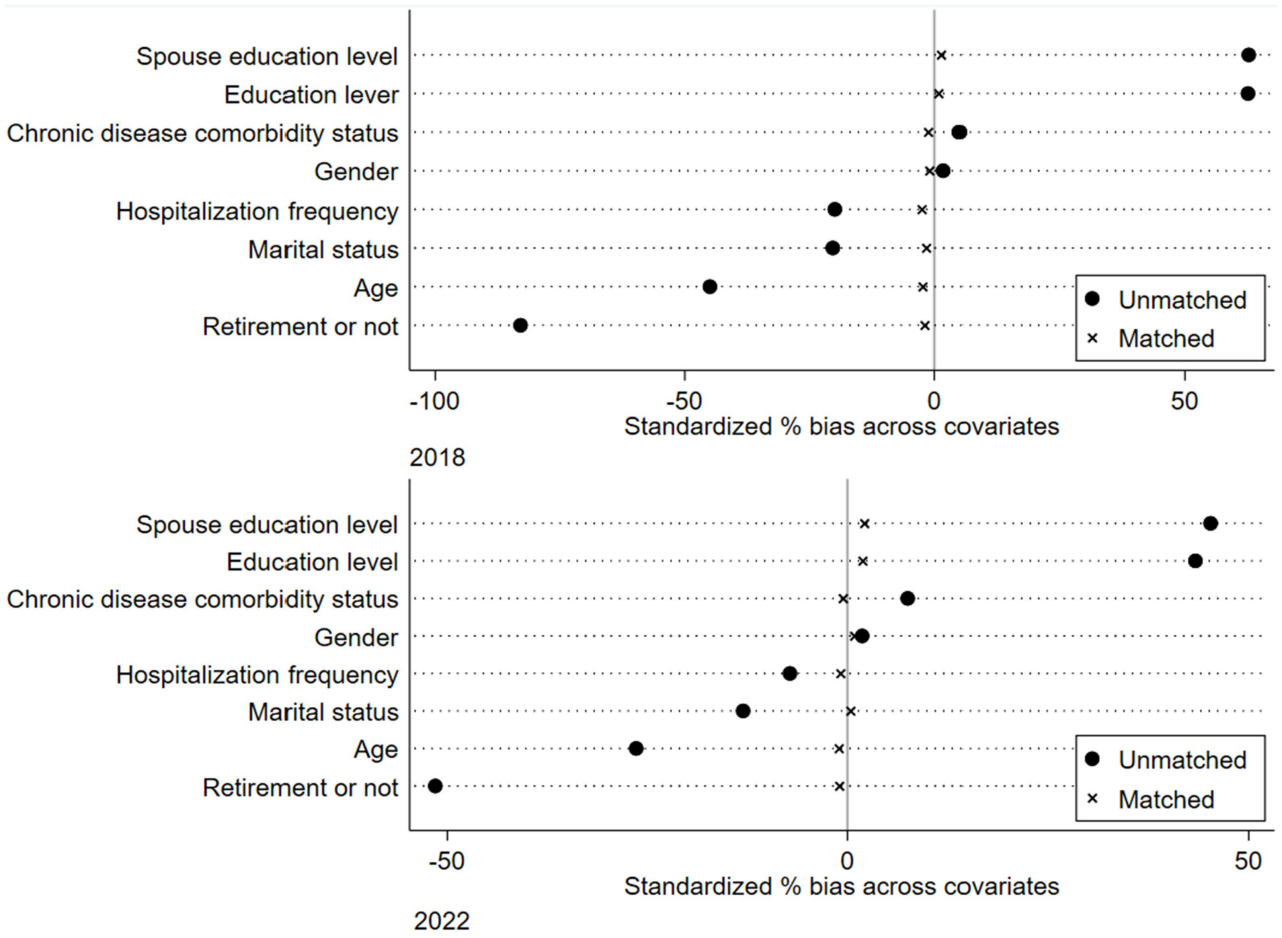
Graphical representation of propensity score matching results for IADL and BADL.

Following the PSM-DID design, Kernel matching was utilized to perform period-by-period matching of the samples, a Logit model was employed to estimate the propensity scores for the samples. Figure 1 displays the differences in control variables before and after matching, indicating a significant reduction in differences between groups post-matching.

### 3.2 Baseline regression results for the effects of physical activity on ADL in older adults

An additional dummy variable, treated, is introduced to differentiate the experimental group from the control group. For the experimental group identified after PSM (i.e., those whose physical exercise status changed from non-participation to participation), treated = 1; for the control group identified after PSM (i.e., those whose exercise participation status remained unchanged), treated = 0. A time dummy variable is set, with time = 0 for the year 2018 and time = 1 for 2022.

Table 3 reports the regression results using PSM-DID, revealing a significantly positive coefficient for the interaction term. This indicates that physical exercise has a positive effect on the IADL and BADL of older adults during the COVID-19 pandemic, with IADL and BADL scores for participants in physical exercise being higher by 0.189 and 0.119 units, respectively, compared to non-participants. This implies that participating in physical activity during the pandemic period had a positive impact on both IADL and BADL.

**Table 3:** Impact of physical exercise on living abilities: PSM-DID estimation results.

	IADL	BADL
	(1)	(2)
t*treated		
	0.189***	0.119**
	(4.05)	(2.86)
Control variables	Yes	Yes
Individual effects	Yes	Yes
Time effect	Yes	Yes
Constant term		
	20.355***	26.777***
	(7.92)	(8.28)
sample size	14,589	14,589

Values in parentheses are *t*-statistics.

***, **, and * indicate significance at the 0.1%, 1%, and 5% levels, respectively.

### 3.3 Age heterogeneity results for the effects of physical activity on ADL in older adults

As age increases, the physical functions of the older adults decline, leading to higher rates of disability, particularly a sharp decrease in self-care abilities among those aged 75 and older. Engaging in appropriate self-care and physical exercise activities can help mitigate the trend of disability in advanced age (23). This paper uses 75 years as a threshold to investigate whether the impact of physical exercise on IADL and BADL differs above and below this age during the pandemic.

The regression results in column (1) of Table 4 indicate that among older adults ‘under 75 years old’, those who engage in physical exercise have an IADL score 0.011 units lower than those who do not, but this effect is not statistically significant. In contrast, for older adults ‘75 years and above’ [column (2) of Table 5], those who engage in physical exercise have an IADL score 0.458 units higher than those who do not, with this effect being significant at the 0.1% level. The regression results in column (3) of Table 4 show that among older adults ‘under 75 years old’, those who engage in physical exercise have a BADL score 0.009 units lower than those who do not, but this effect is not statistically significant. For older adults 75 years and above [column (4) of Table 4], those who engage in physical exercise have a BADL score 0.29 units higher than those who do not, with this effect being significant at the 1% level.

**Table 4:** Heterogeneity analysis.

	IADL		BADL	
	Age < 75	Age ≥ 75	Age < 75	Age ≥ 75
	(1)	(2)	(3)	(4)
t*treated				
	−0.011	0.458***	−0.009	0.290**
	(−0.32)	(4.08)	(−0.28)	(2.95)
Control variables	Yes	Yes	Yes	Yes
Individual effects	Yes	Yes	Yes	Yes
Time effect	Yes	Yes	Yes	Yes
Interaction effects of province and year	Yes	Yes	Yes	Yes
Constant term				
	19.867***	22.505***	18.279	34.103**
	(57.23)	(22.99)	(1.96)	(2.63)
Sample size	9,046	5,543	9,046	5,543

Values in parentheses are *t*-statistics. ***, **, and * indicate significance at the 0.1%, 1% and 5% levels, respectively; columns (1)–(4) exclude age as a control variable.

**Table 5:** Mechanism analysis.

	Mediating effects of retirement on IADL			Mediating effects of retirement on BADL		
	(1)	(2)	(3)	(4)	(5)	(6)
	IADL	Retirement	IADL	BADL	Retirement	BADL
t*treated						
	0.189***	−0.018**	0.196***	0.119**	−0.018**	0.123**
	(4.05)	(−2.86)	(4.17)	(2.86)	(−2.86)	(2.94)
Retirement (Retired)						
			0.406**			0.216**
			(2.85)			(2.05)
Control variables	Yes	Yes	Yes	Yes	Yes	Yes
Individual effects	Yes	Yes	Yes	Yes	Yes	Yes
time effect	Yes	Yes	Yes	Yes	Yes	Yes
Interaction effects of province and year	Yes	Yes	Yes	Yes	Yes	Yes
Constant term						
	20.355***	1.248**	20.254***	27.777**	1.248**	26.723***
	(7.92)	(2.94)	(7.88)	(8.28)	(2.94)	(8.28)
Sample size	14,589	14,589	14,589	14,589	14,589	14,589

Values in parentheses are *t*-statistics. ***, **, and * indicate significance at the 0.1%, 1%, and 5% levels, respectively.

### 3.4 Mechanistic analysis of the effects of physical activity on ADL in older adults

The baseline model demonstrates that engaging in physical exercise leads to a notable increase in IADL and BADL among older adults. This section attempts to further analyze the mechanisms through which physical exercise affects IADL and BADL in the older adults.

Empirical model-based regression results, as shown in columns (1) and (3) of Table 5, indicate that older adults who did not participate in physical exercise in 2018 but did in 2022 reported self-rated health higher by 0.060 units. Furthermore, according to the regression results in column (2) of Table 5, each one-unit increase in self-rated health results in a 0.541 unit increase in IADL. According to the results in column (4) of Table 5, each one-unit increase in self-rated health results in 0.611 units increase in BADL.

When the retirement variable is not included, physical exercise is positively and significantly correlated with both the IADL and BADL of the older adults. When both physical exercise participation and retirement are included simultaneously, the impact coefficients of physical exercise on IADL and BADL increase from 0.189 and 0.119 to 0.196 and 0.123 respectively, and both are significant. The indirect effect of physical exercise on IADL through the nature of retirement is −0.007 (−0.018 × 0.406), which is opposite in sign to the direct effect coefficient (0.196). The indirect effect of physical exercise on BADL through the nature of retirement is −0.004 (−0.018 × 0.216), which is opposite to the direct effect coefficient (0.123). According to methods outlined by scholars regarding mediation and suppressing effects (24), it is evident that the indirect effects of retirement on IADL or BADL are characterized as “suppressing effects,” a type of “mediation effect” also known as inconsistent mediation (25). That is, the masking effect. The effect values are 0.036 = |−0.007/0.196| and 0.033 = |−0.004/0.123| respectively. How to understand this masking effect?

**Figure 2:**
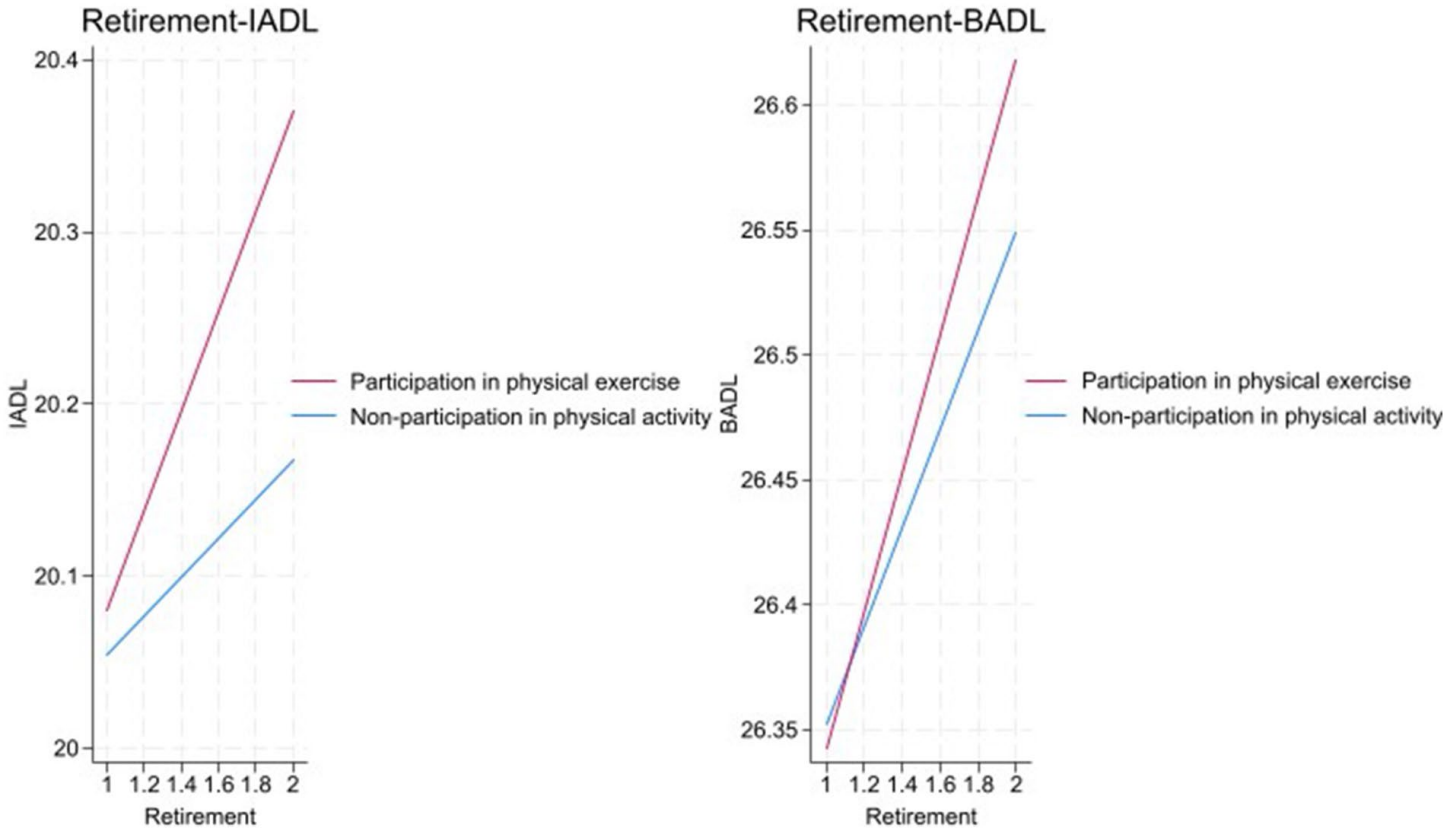
The influence of retirement on IADL and BADL.

The fitting curves of retirement-IADL and retirement-BADL in Figure 2 show that the slope of the fitting line for older adults who participate in physical exercise is greater than that for those who do not. Compared with older adults who do not participate in physical exercise, retirement has a greater impact on the IADL and BADL of older adults who participate in physical exercise. That is, retirement expands the differences in IADL and BADL between the two groups. Thus, without controlling for the nature of retirement, the differences in IADL and BADL due to physical exercise participation are masked. Once the nature of retirement is controlled, the impact of physical exercise on IADL and BADL is amplified.

### 3.5 Model robustness test

This section primarily considers the robustness of the baseline regression results. Previous regression models only controlled for individual and annual fixed effects, yielding results that represented merely the additive effects of individual characteristics and time. Given that various provinces and cities have implemented distinct measures for pandemic control, which inherently differ due to regional development conditions, there is an unavoidable omission of external policy influences that vary by region and time. To mitigate the impact of these external policy variables over time, regional-time interaction terms were included in the model. The results, as shown in Table 6, indicate that the coefficient for t*treated remains significantly positive, confirming that the robustness check has been passed.

**Table 6:** Model robustness test results.

	IADL	BADL
	(1)	(2)
t*treated		
	0.331***	0.250***
	(6.50)	(5.32)
Control variables	Yes	Yes
Year effect	Yes	Yes
Individual effect	Yes	Yes
Province-year interaction effect	Yes	Yes
Constant term		
	21.195***	27.355***
	(8.28)	(8.51)
Samples	14,589	14,589

Values in parentheses are t-statistics; ***, **, and * indicate significance at the 0.1%, 1% and 5% levels, respectively.

## 4 Discussion

The current investigation reveals three critical findings regarding the relationship between physical exercise and ADL in older adults during the COVID-19 pandemic. First, during the pandemic, engagement in physical exercise demonstrated a statistically significant positive impact on both IADL and BADL. Secondly, heterogeneity in these effects was observed around the age of 75 years. Specifically, for individuals aged 75 years and above, participation in physical exercise exerted a significant positive impact on both IADL and BADL. In contrast, among those under 75 years of age, no significant effects of physical exercise on IADL or BADL were detected. Thirdly, retirement status emerged as a significant masking variable that amplifies observed differences in ADL outcomes when controlled analytically. These findings align with and extend previous research across multiple disciplines while introducing novel insights into pandemic-specific geriatric health dynamics.

Our results demonstrate that participating in physical exercise during the pandemic can improve IADL and BADL in older adults. A study that shares our viewpoint documented a 27% reduction in ADL dependency through structured exercise interventions during pandemic restrictions, particularly emphasizing the preservation of lower-limb strength (26). During the COVID-19 pandemic, most older adults reported an increase in sedentary behavior due to limitations on leaving their home and increased free time to pursue seated hobbies (e.g., reading, knitting, tv) (27). Those who adapted their routines to include home-based exercises or virtual classes reported better maintenance of their functional abilities compared to those who remained sedentary (28). Notably, one study confirms that blockades appear to primarily affect the mental health of older adults (29), which may be a result of social distance and isolation (30). An 8-year longitudinal study involving 14,589 older adults individuals in China found that regular physical exercise effectively improved the IADL and alleviated depressive symptoms in the older adults (31). During the pandemic lockdown period, physical exercise can also have a positive contribution by alleviating anxiety symptoms in older adults (32). Participation in physical exercise can divert the attention of older adults and improve cognitive function (33). Physical health, cognitive function, and depression can all affect the ADL of older adults (34). In addition, physical exercise has a positive effect on the cardiovascular and respiratory systems (35), which can better improve the resistance to COVID-19. Therefore, participating in physical exercise during the epidemic can also improve the IADL and BADL of the older adults.

Moreover, the positive impact of physical exercise on IADL and BADL is pronounced in individuals aged 75 and over during the pandemic. In contrast, among those under 75 years of age, no significant effects of physical exercise on IADL or BADL were detected. A longitudinal study on the older adults showed that age is associated with an exacerbation of functional decline, and this age-related decline becomes more pronounced with increasing adversity (36). In 2020, the older adults experienced the COVID-19 pandemic, which exacerbated their functional decline. The older they were, the more obvious the functional decline became. Physical exercise can enhance physical health and slow down functional decline. However, as they age, the older adults gradually reduce their daily physical activities (37), and reported that the older old were more sedentary than the younger old (38). Therefore, compared with the older adults under 75 years old, those 75 years old and above engage in less daily physical activities and rely more on physical exercise to improve their IADL and BADL.

Retirement exerts a masking effect on the impact of physical exercise on the IADL and BADL of the older adults. This masking effect is manifested in the fact that, compared with the older adults who do not engage in physical exercise, retirement has a greater impact on the IADL and BADL of those who do. Retirement is negatively correlated with the IADL and BADL of the older adults. A longitudinal study indicates that complete retirement leads to a 5–16 percent increase in difficulties associated with mobility and daily activities, over an average post-retirement period of 6 years (39). Secondly, after retirement, due to the lack of physical and cognitive activities brought about by work, the muscle strength and cognitive function of the older adults may decline more rapidly (40). This could also be an important factor contributing to the decline in the IADL and BADL of the older adults. However, the impact of retirement appears to be more pronounced among older adults who engage in regular physical exercise compared to those who do not participate in such activities. This phenomenon can be explained by the “survivor effect,” which means that individuals with poor health may be excluded from the survey due to older adults death or serious illness, resulting in the survivor group showing a better state (41). Especially during the COVID-19 pandemic, the older adults are at a higher risk of severe illness and death when infected with the virus (7), so this “survivor effect” is more prominent. Older adults who do not engage in physical exercise are more likely to die due to their lower initial health levels, while the surviving individuals have higher health levels. As a result, retirement has a relatively greater negative impact on the IADL and BADL of older adults who participate in physical exercise.

ADL in the older adults are not static but highly dynamic and diverse, and they can show a degree of reversibility. This means that even those with disabilities might remain independent ADL capabilities (42). Consistent with the findings of this study, most research has demonstrated that physical exercise enhances ADL in the older adults (11, 12, 43). There exists a “dose–response” relationship between exercise and ADL, suggesting that moderate-intensity physical exercise lasting 31 to 59 min per session, three to five times a week, over a period of 6 months or more, is particularly beneficial for enhancing daily living skills (44). The “Physical Activity Guidelines for Americans” recommend that older adults engage in various types of physical activities, including balance training, and aerobic and muscle-strengthening activities, to maintain or improve ADL (45). A lack of aerobic and muscle-strengthening activities can lead to a decline in ADL (46).

Although extensive research has evaluated the impact of physical exercise on older adults ADL, few studies have focused on the role of physical exercise under the constraints of the COVID-19 pandemic. The PSM-DID approach provides robust control for pandemic-specific confounders, addressing limitations in prior observational studies. However several limitations of this study should be acknowledged. First, it did not control for other potential psychological and physiological factors that could affect IADL and BADL. Large-scale surveys have often lacked comprehensive data on physical exercise, such as its intensity, duration, and frequency. Second, the data structure consisted of unbalanced panel data, which may lead to estimation biases due to missing data or an insufficient number of observations. Last, the assessment method for the dependent variables, IADL and BADL, which relied on self-reported questionnaires completed by older adults participants rather than clinical diagnoses, possibly leading to inaccuracies in these assessments. Future research could improve the reliability of findings by incorporating psychological and physiological variables, refining details related to physical exercise, employing balanced panel data, and optimizing methods for evaluating IADL and BADL. Additionally, this study focuses on Chinese older adults. Due to differences in national epidemic control measures and ethnic diversity, the findings may not be generalizable to all older adults populations, particularly those in Western countries. Future research could extend investigations to other countries or ethnic groups.

## 5 Conclusion

Our study employed a PSM-DID model to examine the effects of physical exercise on older adults’ IADL and BADL during the pandemic. During the pandemic, physical exercise still has a positive impact on the IADL and BADL of the older adults. The older adults aged 75 and above are more reliant on physical exercise to improve their IADL and BADL. Retirement amplifies the impact of physical exercise on the IADL and BADL of the older adults. Due to the survivor effect, the relative negative effect of retirement on the IADL and BADL of the older adults who engage in physical exercise is more pronounced.

During the pandemic, the older adults should still engage in physical exercise to improve their activities of daily living, especially those aged 75 and above, as well as retirees. Considering the special nature of the pandemic, first of all, home-based exercises should be encouraged, and online instructional courses should be provided. Secondly, when doing outdoor activities, it is recommended to choose periods with fewer people and open spaces, maintain social distancing, and wear protective gear. At the same time, mental health care should be emphasized. The older adults should be encouraged to maintain social interactions through video calls, and cognitive training should be combined with physical exercise.

## Data Availability

The raw data supporting the conclusions of this article will be made available by the authors, without undue reservation.
