# Corrigendum: Influence of physical exercise on activities of daily living in older adults: an empirical analysis based on propensity score matching and difference-in-differences

**DOI:** 10.3389/fpubh.2025.1622180

**Published:** 2025-06-18

**Authors:** Xiaoni Ma, Xiaotian Li, Li Che, Jie Dong

**Affiliations:** ^1^Graduate School, Capital University of Physical Education and Sports, Beijing, China; ^2^School of Recreation and Community Sport, Capital University of Physical Education and Sports, Beijing, China

**Keywords:** activities of daily living, exercise, COVID-19, difference-in-differences model, propensity score matching, older adults

In the published article, there was an error. The direction of the impact of physical exercise on the IADL and BADL of older adults was incorrectly reported as negative, when it should have been positive.

A correction has been made to Abstract, *Result***s**, this sentence previously stated:

“In the context of COVID-19, participation in physical exercise had a negative impact on the IADL and BADL of older adults. The IADL and BADL of the older adults who participated in physical exercise were 0.189 and 0.119 units lower than those who did not participate in physical exercise. This negative impact also varied by age, for older adults aged 75 years and above, participation in physical exercise exerted a significant positive impact on both IADL and BADL.”

The corrected sentence appears below:

“In the context of COVID-19, participation in physical exercise had a positive impact on the IADL and BADL of older adults. The IADL and BADL of older adults who participated in physical exercise were 0.189 and 0.119 units higher than those who did not participate in physical exercise. This positive impact also varied by age, for older adults aged 75 years and above, participation in physical exercise exerted a significant positive impact on both IADL and BADL.”

The authors apologize for this error and state that this does not change the scientific conclusions of the article in any way. The original article has been updated.

